# Life-Threatening Cardiogenic Shock During Chemotherapy: The Role of Mechanical Ventricular Support in Cardio-Oncology Patients

**DOI:** 10.1016/j.jaccas.2026.107009

**Published:** 2026-02-16

**Authors:** Cristina Aurigemma, Donato Antonio Paglianiti, Francesco Bianchini, Daniela Pedicino, Gianluigi Saponara, Marco Giuseppe Del Buono, Alessia D'Aiello, Tommaso Sanna, Carlo Trani, Francesco Burzotta

**Affiliations:** a Department of Cardiovascular Sciences CUORE, Fondazione Policlinico Universitario A. Gemelli IRCCS, Rome, Italy; b Department of Cardiovascular and Pulmonary Sciences, Università Cattolica del Sacro Cuore, Rome, Italy

**Keywords:** anthracyclines, cardiogenic shock, cardiotoxicity, mechanical circulatory support

## Abstract

**Background:**

Acute cardiogenic shock is a possible and life-threatening complication of anthracycline therapy. Data on the use of percutaneous mechanical circulatory support in this setting remain limited.

**Case Summary:**

A 50-year-old woman developed cardiogenic shock 2 days after receiving epirubicin-based chemotherapy. Owing to progressive hemodynamic instability despite inotropic support, an Impella CP was needed. Extracorporeal membrane oxygenation (ECMO) was avoided given chemotherapy-related pancytopenia and the need to minimize bleeding risk. Progressive hemodynamic recovery was achieved, with normalization of biventricular function at follow-up.

**Discussion:**

This case illustrates acute, reversible anthracycline-induced cardiotoxicity managed with mechanical circulatory support. In thrombocytopenic cardio-oncology patients, a stepwise approach with inotropes and Impella device may represent a safer alternative to ECMO. Early and tailored interventions are key in this high-risk population.

**Take-Home Messages:**

Anthracycline-induced cardiotoxicity may be acute and unpredictable. In cardio-oncology patients with high bleeding risk, an Impella device may represent a safer and effective alternative to ECMO for circulatory support.

**Visual Summary:**
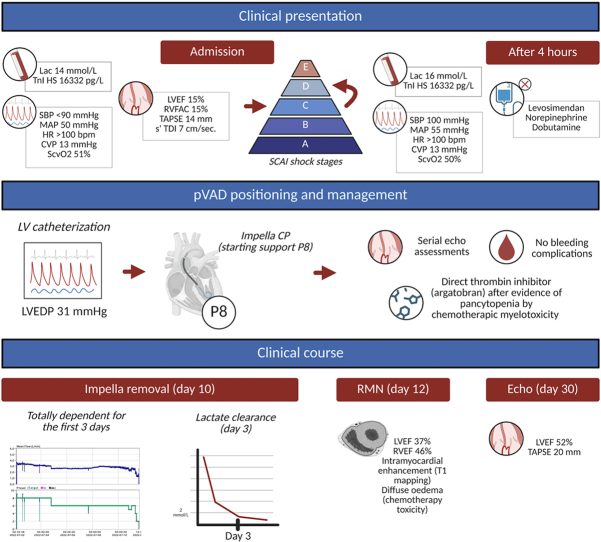
Clinical Course of a Patient With Cardiogenic Shock Due to Chemotherapy-Induced Toxicity The timeline illustrates key events, including initial hemodynamic instability, mechanical circulatory support initiation, therapeutic interventions, and eventual recovery leading to hospital discharge. CVP = central venous pressure; HR = heart rate; Lac = lactate; LV = left ventricle; LVEDP = left ventricular end-diastolic pressure; LVEF = left ventricular ejection fraction; MAP = mean arterial pressure; pVAD = percutaneous ventricular assist device; RMN = magnetic resonance imaging; RVEF = right ventricular ejection fraction; RVFAC = right ventricular fractional area change; SBP = systolic blood pressure; SCAI = Society for Cardiovascular Angiography and Interventions; ScvO_2_ = central venous oxygen saturation; s′ TDI = peak systolic velocity by tissue Doppler imaging; TAPSE = tricuspid annular plane systolic excursion; TnI HS = high-sensitivity troponin I.

## Background

As advancements in cancer therapies extend patient survival, cardiovascular disease and heart failure have emerged as significant causes of morbidity and mortality in this population.^1^ Many cancer treatments, including immune checkpoint inhibitors (ICIs), anthracyclines, and tyrosine kinase inhibitors, have cardiotoxic effects that can lead to acute heart failure and cardiogenic shock.^2^ ICIs can trigger immune-related side effects including myocarditis, which may lead to severe complications and increased mortality. ICI-associated myocarditis can present in various ways, from mild or asymptomatic cases to severe forms that rapidly progress to cardiovascular collapse, life-threatening arrhythmias, or heart failure.^3^

Among conventional chemotherapies, anthracyclines are well-known for causing cardiomyopathy and arrhythmias.^4^ Additionally, 5-fluorouracil is the second-most common agent associated with ischemic complications, arrhythmias, heart failure, and even sudden cardiac death.^5^

For patients experiencing heart failure or cardiogenic shock, acute mechanical circulatory support (MCS) can be used to stabilize hemodynamics as a bridge to recovery or to advanced therapies such as durable MCS or heart transplantation.^6^ Common MCS devices include the intra-aortic balloon pump, Impella, extracorporeal membrane oxygenation (ECMO), TandemHeart, and CentriMag. While ECMO outcomes in cancer patients have been documented,^7^, ^8^, ^9^ data on the use of other MCS devices, such as intra-aortic balloon pump and Impella, remain limited despite their increasing use in managing cardiogenic shock.

This case highlights the growing challenge of chemotherapy-induced cardiogenic shock and the value of a stepwise hemodynamic support approach, with escalation from inotropes to Impella to achieve stabilization while avoiding the potential complications of ECMO.

## History of Presentation

A 50-year-old woman was admitted to our center owing to acute cardiogenic shock. Upon presentation, the patient exhibited severe respiratory distress, fatigue, and rapid clinical deterioration. She developed progressive hypotension (70/40 mm Hg), tachycardia (110-120 beats/min), and oliguria (SCAI [Society for Cardiovascular Angiography and Interventions] shock classification stage C).

## Past Medical History

The patient's medical history included hypertension without prior cardiovascular events. She had no history of diabetes, dyslipidemia, smoking, or family history of cardiovascular disease, and she was not taking any chronic medications other than antihypertensive therapy. Baseline transthoracic echocardiography performed prior to chemotherapy showed normal biventricular size and function, with a left ventricular ejection fraction (LVEF) of 60%. Just 2 days before admission, she received the first cycle of neoadjuvant chemotherapy for breast cancer, which included anthracyclines (epirubicin 90 mg/m^2^) combined with Taxol (paclitaxel) infusion.

## Investigations

**Figure 1:**
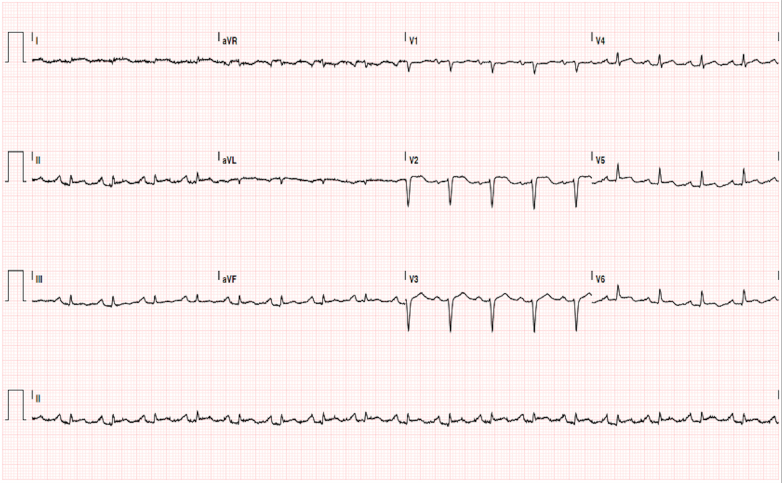
Initial Electrocardiogram Electrocardiogram showing sinus tachycardia, low-voltage QRS complexes, and diffuse ST-segment elevation.

The electrocardiogram showed sinus tachycardia, low-voltage QRS complexes, and diffuse ST-segment elevation (Figure 1). Transthoracic echocardiography revealed severe biventricular dysfunction (LVEF: 15%, right ventricular fractional area change: 15%, tricuspid annular plane systolic excursion [TAPSE]: 14 mm), without significant valvular abnormalities (Video 1). A high lactate level (12 mmol/L) further supported the diagnosis of cardiogenic shock. Laboratory tests revealed markedly elevated high-sensitivity troponin (16,332 ng/L) as well as mild hepatic and renal impairment.

## Differential Diagnosis

Given the acute presentation with ST-segment changes and marked troponin elevation, acute coronary syndrome was excluded by normal coronary angiography. Acute myocarditis was deemed unlikely owing to normal inflammatory biomarkers, absence of fever, and lack of recent respiratory or gastrointestinal infections. Takotsubo syndrome was ruled out given the severe biventricular involvement without the typical regional wall motion pattern or electrocardiographic features. Septic shock was excluded by the absence of fever or clinical signs of infection, normal inflammatory biomarker, and no evidence of a septic source on clinical and laboratory assessment. The absence of echocardiographic signs of acute right ventricular pressure overload, no clinical signs of deep vein thrombosis, and lack of markedly elevated D-dimer levels made acute pulmonary embolism unlikely.

In the context of recent chemotherapy infusion, rapid onset of severe biventricular dysfunction, and myocardial injury, these findings supported the diagnosis of anthracycline- and taxane-induced acute cardiotoxicity.

## Management

Initial treatment included inotropic and vasopressor support with dobutamine, levosimendan, and norepinephrine to stabilize cardiac output and improve systemic perfusion. Coronary angiography was normal, while left heart catheterization revealed an increased left ventricular end-diastolic pressure (31 mm Hg).

However, given persistent hemodynamic instability and inadequate cardiac output despite inotropic therapy (persistent systolic blood pressure: <90 mm Hg, mean arterial pressure: 55 mm Hg, left ventricular outflow tract velocity time integral: 4.2 cm and no lactate clearance, SCAI shock classification stage D), MCS was necessary. Despite the patient's high bleeding risk owing to recent chemotherapy and the right heart compromise (pulmonary artery pulsatility index: 0.9-1.0), an initial strategy of Impella implantation combined with levosimendan was chosen over ECMO to minimize invasiveness.

An Impella CP device (Abiomed) was implanted via the left femoral artery, providing full hemodynamic support (3.0-3.5 L/min at P8-P9 device settings) and was critical in maintaining circulatory stability over the first 72 hours. During her intensive care unit stay, the patient developed progressive pancytopenia, likely due to recent chemotherapy. Consequently, anticoagulation was switched from unfractionated heparin to direct thrombin inhibitor (argatroban) given concerns about chemotherapy-related toxicity and unclear heparin-induced thrombocytopenia risk. After ruling out infectious processes, high-dose corticosteroids were initiated. Following gradual weaning from inotropes and vasopressors, along with complete lactate clearance and hemodynamic stabilization, the Impella device was successfully removed on day 10. No infections or bleeding complications occurred.

## Outcome and Follow-Up

Subsequent cardiac magnetic resonance imaging showed improved biventricular function (LVEF: 37%, right ventricular ejection fraction: 42%) and diffuse myocardial edema consistent with chemotherapy-induced toxicity. The patient had an uneventful recovery and was discharged after 22 days. At 6 months, she underwent breast cancer surgery followed by adjuvant chemotherapy, without complications. At 2 years of follow-up, she remained asymptomatic, and echocardiography confirmed sustained improvement in biventricular function (LVEF: 52%, TAPSE: 20 mm) (Video 2).

## Discussion

**Figure 2:**
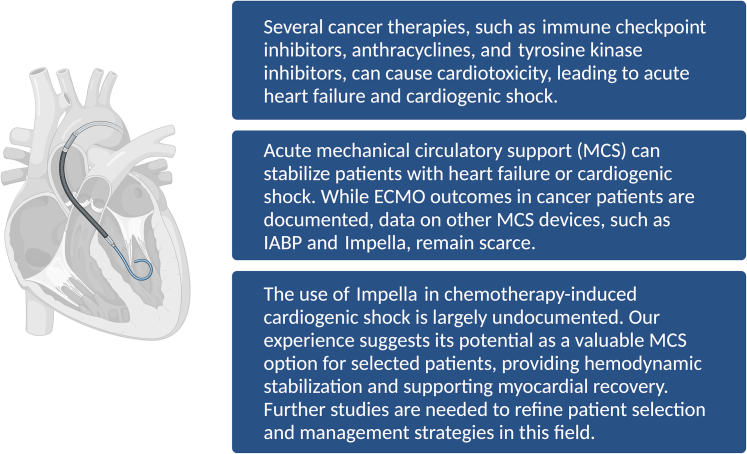
Chemotherapy-Induced Cardiotoxicity and Mechanical Circulatory Support in Cardiogenic Shock Many cancer treatments have cardiotoxic effects that can lead to acute heart failure and cardiogenic shock. Mechanical circulatory support devices, such as IABP, Impella, and ECMO, play a role in stabilizing hemodynamics and supporting myocardial recovery in patients with chemotherapy-related cardiac dysfunction. ECMO = extracorporeal membrane oxygenation; IABP = intra-aortic balloon pump; MCS = mechanical circulatory support.

This case highlights the increasing clinical challenge of chemotherapy-induced cardiogenic shock (Figure 2). Early recognition and prompt intervention, including MCS, are crucial in stabilizing patients and preventing irreversible myocardial damage. Despite initial biventricular dysfunction, a stepwise approach was adopted, prioritizing pharmacological support for right ventricular dysfunction, while opting for the Impella device over ECMO. This decision was based on the need to provide immediate left ventricular unloading and hemodynamic stabilization, while minimizing unnecessary complications associated with ECMO.

The choice of Impella placement was also influenced by the high risk of chemotherapy-induced pancytopenia. Managing anticoagulation in thrombocytopenic patients is particularly challenging, and the smaller thrombotic surface of the Impella device offers more flexible strategies. In contrast, ECMO typically requires aggressive anticoagulation, which increases the bleeding risk. For cardio-oncology patients prone to severe thrombocytopenia, Impella may offer effective support with a safer anticoagulation profile.

Additionally, this case presented as acute fulminant cardiotoxicity, a rare and clinically distinct entity compared with the more familiar chronic, cumulative-dose anthracycline cardiomyopathy. Unlike chronic forms, typically characterized by progressive fibrosis, the diagnosis was supported by cardiac magnetic resonance imaging showing diffuse myocardial edema, indicative of acute myocardial injury and inflammation. This underscores the importance of recognizing severe cardiotoxic reactions that can occur even after a single administration of anthracyclines. The complete recovery of biventricular function demonstrates that this form of cardiotoxicity can be reversible and supports an aggressive early management strategy, including short-term MCS, to bridge patients through the life-threatening initial phase.

After recovery, a further challenge is whether potentially cardiotoxic agents can be safely reintroduced. Available evidence is limited, and current cardio-oncology guidelines^10^ suggest that rechallenge may be considered in selected patients with complete recovery of ventricular function after multidisciplinary evaluation, when the oncologic benefit outweighs the cardiovascular risk. In our patient, adjuvant chemotherapy was resumed several months later after multidisciplinary discussion, under optimized guideline-directed heart failure therapy. Given the complete normalization of biventricular function and the need to ensure optimal cancer control, rechallenge was performed under strict clinical and echocardiographic surveillance and was well tolerated, with no recurrence of cardiac dysfunction. Although the risk of recurrent cardiotoxicity remains non-negligible, chemotherapy rechallenge may be feasible in highly selected patients, provided that rigorous monitoring and shared decision-making guide the therapeutic strategy. In our case, all management decisions were made within a dedicated multidisciplinary shock team, including intensivist and interventional cardiologists, anesthesiologists, cardiac surgeons, and the direct involvement of oncology specialists given the underlying malignancy.

As there is limited evidence on the use of percutaneous left ventricular assist devices such as the Impella in chemotherapy-induced cardiogenic shock, this case is particularly relevant. Our experience highlights its potential role in selected patients as a valuable MCS option, promoting hemodynamic stabilization and myocardial recovery. Future studies are needed to define patient selection criteria and optimal management strategies for this emerging field.

## Conclusions

This case underscores the critical role of multidisciplinary strategy in managing chemotherapy-induced cardiogenic shock, balancing the benefits of MCS with the risks of thrombotic and vascular complications. For these critically ill patients requiring intensive care, therapeutic options are limited and do not alter disease progression. Despite the expanding field of cardio-oncology, prospective data in this high-risk population remain limited. There is a need for preventive treatments aimed at reducing cardiac damage, and the increasing use of MCS now provides a viable treatment option for patients with acute heart failure.

## Funding Support and Author Disclosures

Dr Aurigemma has received speaker fees from Abbott, Abiomed, Medtronic, Edwards Lifesciences, and Daiichi Sankyo. Dr Trani has received speaker fees from Abbott, Abiomed, Medtronic, Boston Scientific, and Daiichi Sankyo. Dr Burzotta has received speaker fees from Abbott, Abiomed, Medtronic, and Edwards Lifesciences. All other authors have reported that they have no relationships relevant to the contents of this paper to disclose.

**Take-Home Messages**

• Anthracycline-induced cardiotoxicity may be acute and unpredictable. Early recognition and tailored management are critical to enable full recovery.
• In cardio-oncology patients with high bleeding risk, the Impella device may represent a safer and effective alternative to ECMO for circulatory support.

## Appendix

Video 1 Parasternal Short-Axis and Apical 4-Chamber View Echocardiogram Initial transthoracic echocardiography revealing severe biventricular dysfunction (LVEF: 15%, right ventricular fractional area change: 15%, TAPSE: 14 mm), without significant valvular abnormalities.

Video 2 Follow-Up Apical 4-Chamber View Echocardiogram Follow-up transthoracic echocardiography showing improvement in biventricular function (LVEF: 52%, TAPSE: 20 mm).
